# *Chlorella mirabilis* as a Potential Species for Biomass Production in Low-Temperature Environment

**DOI:** 10.3389/fmicb.2013.00097

**Published:** 2013-04-23

**Authors:** S. P. Shukla, J. Kvíderová, J. Tříska, J. Elster

**Affiliations:** ^1^Aquatic Environment Management Division, Central Institute of Fisheries EducationMumbai, India; ^2^Centre for Phycology, Institute of Botany AS CRTřeboň, Czech Republic; ^3^Faculty of Science, University of South BohemiaČeské Budějovice, Czech Republic; ^4^Laboratory of Metabolomics and Isotopic Analyses, Global Change Research Centre AS CRČeské Budějovice, Czech Republic

**Keywords:** microalgae, growth rate, N and C manipulation, fatty acid content, low temperature

## Abstract

Successful adaptation/acclimatization to low temperatures in micro-algae is usually connected with production of specific biotechnologically important compounds. In this study, we evaluated the growth characteristics in a micro-scale mass cultivation of the Antarctic soil green alga *Chlorella mirabilis* under different nitrogen and carbon sources followed by analyses of fatty acid contents. The micro-scale mass cultivation was performed in stable (in-door) and variable (out-door) conditions during winter and/or early spring in the Czech Republic. In the in-door cultivation, the treatments for nitrogen and carbon sources determination included pure Z medium (control, Z), Z medium + 5% glycerol (ZG), Z medium + 5% glycerol + 50 μM KNO_3_ (ZGN), Z medium + 5% glycerol + 200 μM NH_4_Cl (ZGA), Z medium + 5% glycerol + 1 mM Na_2_CO_3_ (ZNC), Z medium + 5% glycerol + 1 mM Na_2_CO_3_ + 200 μM NH_4_Cl (ZGCA) and Z medium + 5% glycerol + 1 mM Na_2_CO_3_ + 50 μM KNO_3_ (ZGCN) and were performed at 15°C with an irradiance of 75 μmol m^−2^ s^−1^. During the out-door experiments, the night-day temperature ranged from −6.6 to 17.5°C (daily average 3.1 ± 5.3°C) and irradiance ranged from 0 to 2,300 μmol m^−2^ s^−1^ (daily average 1,500 ± 1,090 μmol m^−2^ s^−1^). Only the Z, ZG, ZGN, and ZGC treatments were used in the out-door cultivation. In the in-door mass cultivation, all nitrogen and carbon sources additions increased the growth rate with the exception of ZGA. When individual sources were considered, only the effect of 5% glycerol addition was significant. On the other hand, the growth rate decreased in the ZG and ZGN treatments in the out-door experiment, probably due to carbon limitation. Fatty acid composition showed increased production of linoleic acid in the glycerol treatments. The studied strain of *C. mirabilis* is proposed to be a promising source of linoleic acid in low-temperature-mass cultivation biotechnology. This strain is a perspective model organism for biotechnology in low-temperature conditions.

## Introduction

Micro-algae are considered to be a promising alternative in aquaculture feed formulation due to their photoautotrophic mode of nutrition and their ability to grow in inorganic media under a wide range of environmental conditions (e.g., Bennemann et al., 1987). Two major advantages associated with the use of unicellular organisms including micro-algae are: (a) a well-defined protocol for industrial production under controlled and environmentally safe conditions (Sanchez et al., 1995) and (b) the inherent capability for manipulating (physiological and genetic) the strains to produce higher quantities of the desired compounds (e.g., poly-unsaturated fatty acids, polyols, carotenoids, or phycocyanin (Tan and Johns, 1991; Day and Tsavalos, 1996).

Substantial information about the applications of micro-algae from various bio-geographic regions is available in the literature (Zhou et al., 1991; Langden and Onal, 1999; Harel et al., 2002, etc.). However, there is a scarcity of base-line data for the potentialities of micro-algae originating from various habitats in the polar regions. Cold-adapted, psychrophilic, or psychrotolerant, micro-algae have evolved a complex network of adaptation/acclimatization reactions to survive in low-temperature environments (Shukla et al., 1997a,b; Shukla and Kashyap, 2003; Elster and Benson, 2004; Pandey et al., 2004; Vonshak and Torzillo, 2004; Elster et al., 2008). Their successful survival is usually connected with production of specific biotechnologically important compounds like poly-unsaturated fatty acids, polyols, and low-temperature enzymes. Especially poly-unsaturated fatty acids are of primary interest to the food industry and could be obtained from various micro-algae and cyanobacteria (řezanka et al., 2008, 2009). Since these compounds are valuable in the food, cosmetics, and pharmacological industries, such biotechnology in low-temperature environments could be beneficial in areas with lower temperatures like temperate regions in winter/early spring conditions or the polar regions in summer.

So far, the majority of commercial mass cultivations were performed in tropical and subtropical zones due to growth requirements of commercially used slightly thermophilic and mesophilic strains like *Spirulina*, *Dunaliella salina*, or *Haematococcus pluvialis* (Borowitzka, 2005). In temperate areas, because of requirements for higher and more or less stable temperatures, and because of appropriate light conditions, out-door mass cultivations operate during summer months (May–September) only (Masojídek et al., 1999). The cultivation units do not operate during the rest of the year. Cultivation of cold-adapted strains in late winter or spring (February–May) could extend unit utilization for approximately 4 months, providing additional income. In polar regions, to the best of our knowledge, micro-algae and cyanobacteria mass cultivation technology has not been tested yet. Polar summer conditions offer continuous light and more/less stable temperature conditions which provide an additional advantage. The appropriate conditions for mass cultivation of low-temperature adapted micro-algae and cyanobacteria in polar areas could be proposed for three or at a maximum 4 months only.

Even though the cultivation scale-up of micro-algae in polar regions seem to be difficult due to the harsh climatic conditions, on the basis of introduced results the innovative development of micro-algae biotechnology in low-temperature environment is feasible. It is challenge for micro-algae research society to development photobioreactors for extreme polar regions environmental conditions. Such a technology could potentially help to develop local industry and at the same time provide a viable means of treating domestic wastewaters generated by local communities.

There are two plausible reasons for the limited number of reports on polar micro-algae biotechnological applications; logistical constraints in the collection of samples, and the tedious process of isolation and cultivation of unialgal and axenic strains. A prospective strain for low-temperature mass cultivation should be able to grow at near-zero temperatures (0–5°C), tolerate temperatures up to 20°C, have minimum requirements for nutrient additions and high primary production rates across a broad temperature range. The ecophysiological requirements for temperature and light of *Chlorella*-like species isolated from the Arctic or Antarctic could be suitable for out-door cultivation under varying conditions. Generally, polar micro-algae should be adapted to low nutrient conditions (Elster and Svoboda, 1995; Elster, 1996, 2002; Dickson, 2000; Elster et al., 2001; Elster and Benson, 2004; Kaštovská et al., 2005); such a lower nutrient demand can reduce the costs of mass cultivation. A nutrient requirement study confirmed the low nitrogen requirements in some Arctic and Antarctic strains, but also revealed serious carbon limitation during cultivation (Shukla et al., 2011).

The aim of this study was to evaluate the possibilities of micro-scale mass cultivation (growth rate) of an Antarctic soil green alga *Chlorella mirabilis* strain L10 in in-door and out-door conditions for production of poly-unsaturated fatty acids under different nutrient conditions. The period of out-door cultivation, performed in a temperate region – the Czech Republic, Central Europe, was selected as late winter or early spring (February–April) in order to mimic the temperature environments of the polar summer. The results of the study serve as base-line information for the optimization of biomass yield and its biotechnological composition in a polar strain of micro-algae under in-door and out-door conditions, and potentially also for out-door cultivation of micro-algae in polar environments.

## Materials and Methods

### Experimental strain

The experimental strain *Chlorella mirabilis* Andreeva 1973 strain Lukešová 10/1997 (L10) was isolated from deglaciated soil on King George Island, South Shetland, maritime Antarctic (S 62° 10′ W 58° 30′). The strain is kept in the Culture Collection of Autotrophic Organisms at the Institute of Botany AS CR in Třeboň, Czech Republic. The stock culture was kept at 12°C and 50 μmol m^−2^ s^−1^ of photosynthetically active radiation (PAR) in Z medium (Staub, 1961). The growth optimum and limits of the experimental strain were evaluated using cultivation in crossed gradients of temperature (−4 to +24°C) and PAR (fluorescent tubes; 5–65 μmol m^−2^ s^−1^). The cultivation unit and procedures are described in Kvíderová and Lukavský (2001) and Kvíderová and Lukavský (2005). The minimum growth temperature of this strain is 4.5–10.1°C and the maximum >20.5°C. Optimum growth was observed in the range 10.1–20.5°C. The minimum irradiance requirement is 12.3–15.9 μmol m^−2^ s^−1^, maximum at 27.8 μmol m^−2^ s^−1^, with an optimal range of 15.9–21.5 μmol m^−2^ s^−1^. Tolerance to irradiances above 65 μmol m^−2^ s^−1^ remains unknown, since these values were not used during cultivation in crossed gradients.

### Mineral media composition

Sterilized Z medium (2 l) with the desired concentrations of chemicals was added to each cultivation treatment. Addition of 5% (v/v) glycerol was used: (a) to prevent freezing of the suspension in the out-door experiment, since sub-zero temperatures were expected and (b) as an organic C source. Glycerol, together with the electrically heated mattresses of the cultivation platform bottom (see description of the out-door cultivation unit below), kept the cultivation solution in a liquid state. The effect of different nitrogen sources was tested by addition of either 200 μM NH_4_Cl or 50 μM KNO_3_. To test and prevent carbon limitation, 1 mM Na_2_CO_3_ was added. The combined effect of nitrogen and carbon additions was also evaluated (see detailed treatment descriptions and their abbreviations in Table 1). pH of the complete medium was adjusted to 7.5 by titration of 0.1 N HCl and NaOH. The pH of the medium was checked by a CPH51 laboratory pH-meter (Crytur, Czech Republic).

**Table 1:** **Nutrient treatments, their abbreviations and applications in in-door and out-door micro-scale mass cultivation experiments**.

Treatment	Abbreviation	In-door	Out-door
Z medium (control)	Z	✓	✓
Z medium + 5% (v/v) glycerol	ZG	✓	✓
Z medium + 5% (v/v) glycerol + 200 μM NH_4_Cl	ZGA	✓	
Z medium + 5% (v/v) glycerol + 50 μM KNO_3_	ZGN	✓	✓
Z medium + 5% (v/v) glycerol + 1 mM Na_2_CO_3_	ZGC	✓	✓
Z medium + 5% (v/v) glycerol + 1 mM Na_2_CO_3_ + 200 μM NH_4_Cl	ZGCA	✓	
Z medium + 5% (v/v) glycerol + 1 mM Na_2_CO_3_ + 50 μM KNO_3_	ZGCN	✓	

Nutrient treatments for the out-door conditions were selected according to the best growth parameters tested in the in-door experiments. Out-door cultivation conditions are summarized in Table 1.

### Growth rate calculation

The inoculum sizes of an exponentially growing population of *Chlorella mirabilis* strain Lukešová L10 were added to each flask or well in order to obtain initial optical density values (absorbance) at 750 nm (*A*_750_) of 0.05. This *A*_750_ value corresponds to the detection limit of the iEMS plate reader (LabSystems, Finland; see, Kvíderová, 2010 for initial cell density justification). The *A*_750_ was measured once every 2 days during the in-door cultivation and once a day during the out-door cultivation. Six 200 μl sub-samples were poured into individual wells in a micro-plate (transparent, 96 wells, flat bottom) and the *A*_750_ was measured using a plate reader.

The values of the *A*_750_ were converted to number of cells *N* (cells ml^–1^) according to a conversion equation. The conversion equation parameters were estimated according to the method described in Kvíderová and Lukavský (2003) and Kvíderová (2010). The culture was grown in similar conditions as those in the experiments. The density of the undiluted culture was set to *A*_750_ = ∼1.5–2.0 and was diluted by factors of 0.5, 0.3, 0.1, 0.05, 0.03, 0.01, 0.005, 0.003, 0.001, 0.0005, 0.0003, and 0.0001. Distilled water served as a blank. Cell number was counted using Bürker’s chamber. The conversion equation for *Chlorella mirabilis* L10 and the above specified equipment (plate reader, micro-plate, suspension volume) is as follows (*r^2^* = 0.995):

$$N\;\left[\text{cells}\;\text{m}{\text{l}}^{-1}\right]=\frac{A_{750}-0.04}{3.54\times 10^{-8}}$$

where *N* is the number of cells per milliliter and *A*_750_ the value of optical density (absorbance) at 750 nm.

Two types of growth rates were calculated. The partial growth rate (μ_part_; day^−1^) was calculated as

$$\mu_{\text{part}}\;\left[{\text{d}}^{-1}\right]=\frac{\ln N_{t+\text{SI}}-\ln N_{t}}{\text{SI}}$$

where μ_part_ is the partial growth rate, *N_t_* is the cell count per ml during the first *A*_750_ measurement, *N_t+SI_* is the cell count in the second *A*_750_ measurement after SI, and SI is sampling interval in days, i.e., 2 for the in-door experiment and 1 for the out-door experiment. The growth rate during the whole experiment (μ, day^−1^) was calculated as the slope of the linear regression of the dependency of ln *N* on time.

### In-door micro-scale mass cultivation experiment

The in-door mass cultivation experiment was performed in a closed cultivation unit (Labio, Czech Republic). The cultivation apparatus consisted of a 250 l insulated chamber with outer dimensions of 1,350 × 750 mm and 1,120 mm depth. Refrigerating (150 W) and heating (150 W) units and a ventilator were installed in the bottom part of the box. A temperature range of 0 up to 30°C could be regulated. Above the chamber bottom, a linear shaker plate of size 800 mm × 410 mm × 20 mm with a speed of 0–1/2 oscillations per second was installed. A cover made of double thermal insulation glass closed the upper part of the chamber. The chamber was illuminated by two to five fluorescent tubes positioned above the glass cover.

During the experiment, PAR and temperature in the chamber were 75 μmol m^−2^ s^−1^ and 15°C, respectively. A lower irradiance corresponding to the growth optimum could not be set due to the technical parameters of the unit. The irradiance of 75 μmol m^−2^ s^−1^ corresponds to the minimum number of fluorescent tubes which can ensure homogeneous irradiance inside the cultivation chamber. Seven 3-l Erlenmeyer flasks were installed in the box and each flask was filled with 2 l of Z medium with the desired concentrations of chemicals (see Table 1 for treatment descriptions). The cultures were shaken by a built-in linear shaker in the cultivation chamber. The speed of the shaking rate was kept at a maximum of a 1/2 oscillation per second in order to prevent cell damage. A small aquarium pump pumped fresh air into the box in order to prevent carbon limitation. The in-door experiment lasted 8 days. The *A*_750_ measurements were performed every second day.

### Out-door micro-scale mass cultivation experiment

**Figure 1:**
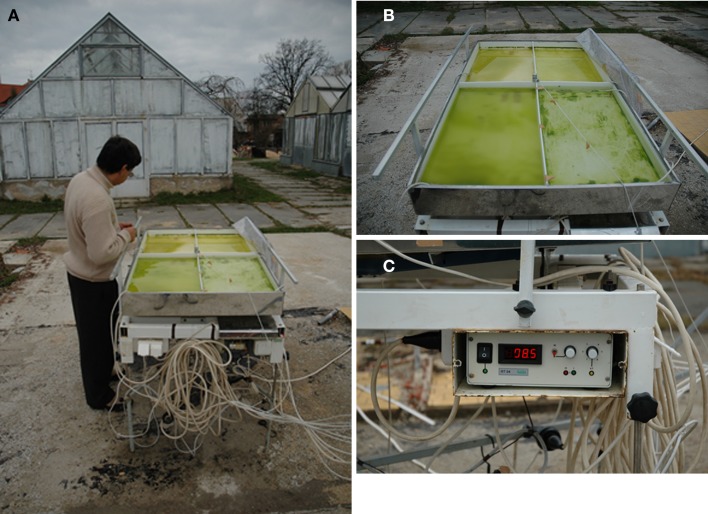
**The out-door micro-scale mass cultivation experiment**. **(A)** The cultivation unit (Labio, Czech Republic), **(B)** detailed view of the cultivation well with micro-algal culture, **(C)** detailed view of the thermoregulation control unit.

The out-door cultivation experiment was performed in an out-door cultivation table (Labio, Czech Republic, Figure 1, see (Elster et al., 2001) for unit technical details). The cultivation unit consisted of a solid platform supporting a shallow polycarbonate pan with dimensions of 1,333 mm × 750 mm and 70 mm deep. The pan was made with a double bottom. In the drawer-like cavity, four electrically heated mattresses (800 W each) were installed. The pan area was divided into four rectangular chambers of equal size using a Perspex sheet. The pan was gently shaken using a motor, worm screws and belt pulley. To each rectangular well on the cultivation unit, 2 l of Z medium with the desired concentrations of chemicals was added (see Table 1 for treatment descriptions). The platform was covered with a thin, transparent polythene sheet to prevent loss of the medium due to evaporation and stabilize the temperature of the culture.

The platform was installed in the backyard of the Institute of Botany in Trebon (Figure 1) and three 6-day independent cultivations were performed from March 13 to April 7, 2005. The experiment performed from March 19 to 25, 2005 showed the highest harvest and was selected for a detailed evaluation. Irradiance, temperature, and pH (Kombibox WTE, Weilheim, CB 570) of the cultivation treatments together with regulation of the heating mattresses were performed in the morning and afternoon. In addition, weather conditions (air temperature including min. and max. values, irradiance, air pressure, humidity, and precipitation) were monitored at 15 min intervals by an automatic weather station (AWS; ENVI s.r.o). The station is installed on the roof of a building about 200 m from the place where the experiments were performed.

### Fatty acid analysis

At the end of both the in-door/out-door cultivation experiments, algal biomass was centrifuged and lyophilized. About 25 mg of the lyophilized micro-algae biomass were mixed in a 7 ml vial with 5 ml of a chloroform:methanol (2:1) mixture and kept at 50°C for 2 h with occasional shaking. The extract was separated and the biomass extracted once again by the same procedure. The joint extracts were filtered and evaporated to dryness by nitrogen blowing. About 0.5 ml of boron trifluoride in methanol was added to the remainder and the reaction mixture was kept for several minutes at 50°C. After the reaction was completed, 0.5 ml of water was added and the reaction mixture was extracted three times with hexane. Joint extracts were filtered through a short column filled with anhydrous sodium sulfate, concentrated down to 100 μl by nitrogen flow and 1 μl of the hexane extract was injected into a GC-MS (Finnigan GCQ instrument; column Zebron ZB-5, Phenomenex, USA, 30 m × 0.25 mm × 0.25 μm; temperature program: initial temperature 60°C, followed by a step-wise increase of 20°C min^−1^ to 180°C, then another step-wise increase of 2°C min^−1^ to 275°C; temperature of the transfer line 275°C, ion source 200°C; linear velocity of the carrier gas (helium) 40 cm s^−1^; full scan spectra in the range of relative mass *m*/*z* 50–450).

The acids were measured as methylesters arising from the transesterification reaction using boron trifluoride in methanol. The qualitative analyses of fatty acids methyl esters were performed using external Bacterial Acid Methyl Ester (BAME) Standard Mix (Supelco, Sigma-Aldrich, Czech Republic). Quantification of the methylesters was performed by normalization; the minor peaks which were not present in the BAME mixture were not evaluated. The repeated injection of the mixture of methylesters revealed R.S.D. for individual peaks up to 5% as a maximum.

### Statistics

Statistical analyses were performed using Statistica 10 (StatSoft, USA). Descriptive statistics were used to characterize the weather conditions during the out-door cultivation. The effect of individual treatments on growth rate was evaluated by *t*-test. When individual addition effects were evaluated, the Honest Significant Differences (HSD) test for unequal *n* was used. Correlation analysis evaluated the relationship between the meteorological data obtained from the AWS and the microclimate data measured in the cultivation platform. Correlation analysis was also used to evaluate the effect of individual environmental variables on growth rates in control and manipulated treatments. The results were considered significant for *p* < 0.05.

## Results

### In-door micro-scale mass cultivation experiment

The μ_part_ during the in-door cultivation was relatively low, ranging from −0.02 to 0.18 day^−1^ in the control and from 0.09 to 0.28 day^−1^ in the nutrient treatments and varied during the cultivation (Table 2). The drop in the partial growth rates from Day 4 to Day 6 was caused by technical problems, since it occurred almost in all cultures.

**Table 2:** **μ_part_ and μ (day^−1^; mean ± SD, *n* = 6) of *Chlorella mirabilis* in various compositions of growth medium (see Table 1 for growth media explanations) under in-door conditions (irradiance of 75 μmol m^−2^ s^−1^ and temperature of 15°C)**.

	Growth medium						
	Z	ZG	ZGA	ZGN	ZGC	ZGCA	ZGCN
μ_part 0–2_	0.15 ± 0.08^a^	0.28 ± 0.09^a^	0.23 ± 0.07^a^	0.25 ± 0.04^a^	0.22 ± 0.05^ab^	0.26 ± 0.05^a^	0.17 ± 0.04^a^
μ_part 2–4_	0.12 ± 0.05^a^	0.18 ± 0.04^bc^	0.18 ± 0.06^a^	0.23 ± 0.03^a^	0.27 ± 0.02^ab^	0.18 ± 0.04^ab^	0.17 ± 0.08^a^
μ_part 4–6_	-0.02 ± 0.08^b^	0.11 ± 0.05^c^	0.08 ± 0.02^b^	0.22 ± 0.04^a^	0.16 ± 0.03^a^	0.09 ± 0.08^b^	0.16 ± 0.08^a^
μ_part 6–7_	0.18 ± 0.04^a^	0.22 ± 0.03^ab^	0.25 ± 0.04^a^	0.24 ± 0.04^a^	0.31 ± 0.16^b^	0.23 ± 0.07^a^	0.23 ± 0.02^a^
μ	0.13 ± 0.04	0.23 ± 0.03	0.17 ± 0.00	0.23 ± 0.01	0.22 ± 0.01	0.22 ± 0.03	0.20 ± 0.04

*The μ_part_ was calculated for each period between subsequent *A*_750_ measurements (μ_part 0–2_ for day 0–day 2; μ_part 2–4_ for day 2–day 4; μ_part 4–6_ for day 4–day 6; μ_part 6–8_ for day 6–day 8) and μ for the whole cultivation lasting 8 days. The same letter in superscript indicates homologous groups as recognized by the Tukey HSD test at *p* = 0.05 (*n* = 6 in each group)*.

**Figure 2:**
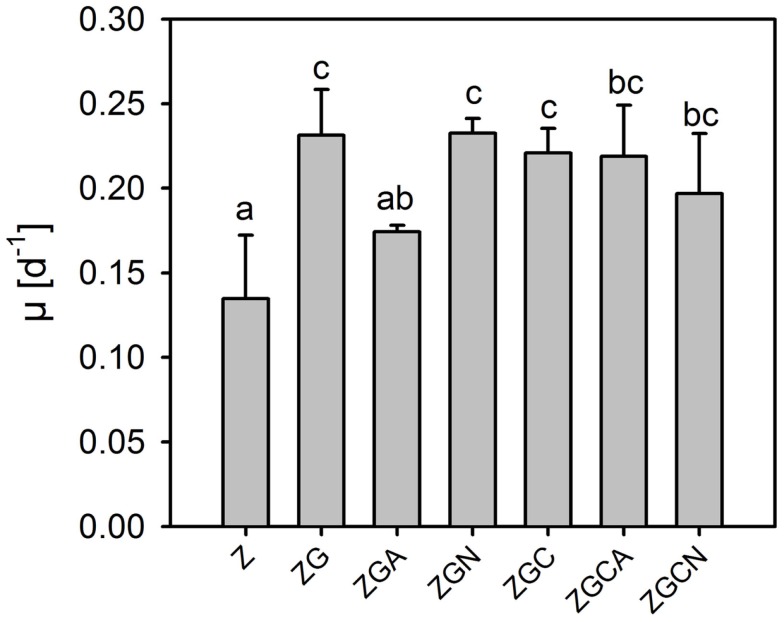
**Relative growth rates (mean ± SD, *n* = 6) of *Chlorella mirabilis* under different nutrient treatments in the in-door micro-scale mass cultivation experiment**. See Table 1 for treatment abbreviations. The same letter indicates homologous groups as recognized by the Tukey HSD test at *p* = 0.05; the Z medium (control) is included in the group labeled “a.”

Even if the whole cultivation duration was considered, the μ of the control and various nutrient treatments were low and did not exceed 0.25 day^−1^ (Table 2, Figure 2). The μ ranged from 0.06 to 0.17 day^−1^ and from 0.16 to 0.28 day^−1^ in the control and nutrient treatments, respectively.

**Figure 3:**
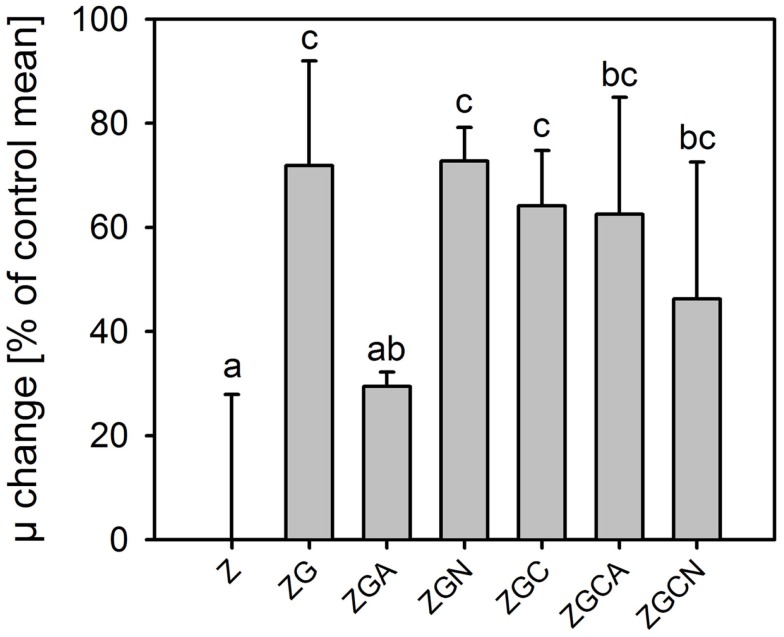
**Change in relative growth rates (mean ± SD, *n* = 6) of *Chlorella mirabilis* under different nutrient treatments in the in-door micro-scale mass cultivation experiment (see Table 1 for treatment abbreviations)**. The values were normalized to control mean (100%). The zero line corresponds to 100% of the control. The same letter indicates homologous groups as recognized by the Tukey HSD test at *p* = 0.05; the Z medium (control) is included in the group labeled “a.”

Relative growth rates were significantly greater in all nutrient treatments (*t*-test, *n* = 6 in each treatment, *p* < 0.049 in all cases; Figure 2). The highest growth stimulations were 72 ± 20% and 73 ± 6% greater compared to the control in the ZG and ZGN treatments, respectively. The minimum increase in growth rate compared to the control was 29 ± 3% in the ZGA treatment, indicating possible worse ammonium utilization (Figure 3). When the individual nutrient addition effects on the growth rate were considered, only glycerol increased the growth significantly (HSD for non-equal *n*, *p* < 0.001).

### Out-door micro-scale mass cultivation experiment

#### Weather and microclimate conditions

**Figure 4:**
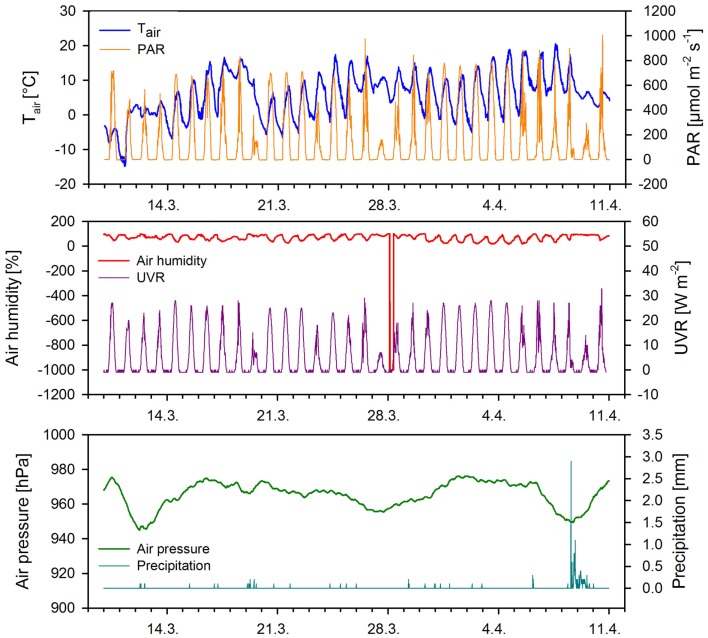
**Meteorological conditions during the out-door cultivation measured at 15-min intervals**. Abbreviations: *T*_air_, air temperature; PAR, photosynthetically active radiation; UVR, ultraviolet radiation.

The weather conditions during the out-door micro-scale mass cultivation (March 19–25, 2005) experiment were relatively stable with several minor precipitation events (Table 3, Figure 4). The mean air temperature during the cultivation was 3.1 ± 5.3 (mean ± SD, *n* = 573). The lowest air temperature was −6.6°C while the maximum was 17.5°C. Mean air pressure was 968 ± 2 hPa (mean ± SD, *n* = 573) with a minimum of 963 hPa and maximum of 973 hPa. Mean global irradiance was 329 ± 238 W m^−2^ (mean ± SD, *n* = 293) and reached even 711 W m^−2^. Mean relative air humidity was 71 ± 20% (mean ± SD, *n* = 573). The minimum relative air humidity was only 23% while the maximum rose to 98% during precipitation events. Seven precipitation events were recorded during the cultivation; however these precipitations were small, not exceeding 0.2 mm. The weather data for each day of the out-door experiment are summarized in Table 3 and these data were used for further evaluation of environmental conditions on *Chlorella mirabilis* primary productivity.

**Table 3:** **Summarized weather conditions during the out-door cultivation experiment (March 19–25, 2005)**.

Date	Time	Air temperature (°C)			Irradiance		Air humidity	Air pressure
		Mean	Min.	Max.	Mean (W m^−2^)	Sum (kJ m^−2^)	Mean (%)	Mean (hPa)
19 March	11:00	4.4	1.8	10.3	89.4	2,578	81.6	969.8
	18:30	
19 March	18:30	−2.7	−5.6	2	0	0	88.2	972.8
20 March	6:15	
20 March	6:30	1.9	−5.9	6.9	505.2	22,316	54.5	971
	18:30	
20 March	18:45	−3	−6.2	3.8	0	0	79.4	968.4
21 March	6:15	
21 March	6:30	3.7	−6.6	9.1	499.1	21,984	44.3	967.3
	18:30	
21 March	18:45	−1.6	−4.7	5.2	0	0	71	967
22 March	6:15	
22 March	6:30	5.4	−5.8	10.1	486	21,500	54.4	965.3
	18:30	
22 March	18:45	1.1	−1.5	6.3	0	0	85.8	965.9
23 March	6:15	
23 March	6:30	7.8	−2	13.1	175.2	7,781	67.5	967.5
	18:30	
23 March	18:45	4.4	−0.8	9.4	0	0	85.9	967.3
24 March	6:15	
24 March	6:30	10.3	−2.1	17.5	439.7	19,294	61.9	966.9
	18:30	
24 March	18:45	4.9	−0.9	12.4	0	0	82.6	965.7
25 March	6:00	
25 March	6:15	3.1	−1	8.4	234	3,188	86.4	965.2
	10:00	

*Presence and/or absence of sun irradiation delimit the time of day and night period for all measured parameters (mean, maximal and minimal air temperature, mean and sum of irradiance, mean air humidity and mean air pressure). For daylight periods, the mean values and sum of irradiance are shown*.

The temperature and PAR in the cultivation unit differed a little from the AWS data (Table 4). The increased temperature in the cultivation unit was probably caused by it being covered by the polyethylene sheet resulting thus in a green-house effect. However, strong positive correlations were found between PAR irradiance in the cultivation unit and the AWS global radiation data (*n* = 17, *r* = 0.917, *t* = 8.93, *p* < 0.001) as well as between the temperature in the cultivation unit and the AWS air temperature (*n* = 17, *r* = 0.668, *t* = 3.48, *p* = 0.003), so the meteorological data could serve as a proxy data for microclimate conditions, with specific respect to the variation during cultivation.

**Table 4:** **Microclimate conditions (temperature and PAR) during the out-door experiment (March 19–25, 2005)**.

	Date	Time	Temperature (°C)					PAR
			Z	ZG	ZGN	ZGC	Mean ± SD	(μmol m^−2^ s^−1^)
Day 0	19 March	11:00	9.9	9.4	9.5	9.5	9.6 ± 0.2	240
		17:00	8.1	7.8	7.5	7.4	7.7 ± 0.3	75
Day 1	20 March	8:30	8.2	7.9	6.8	6.6	7.4 ± 0.8	269
		14:00	25.1	24.7	25	24.8	24.9 ± 0.2	1,040
		16:50	14.1	13.9	13.9	13.8	13.9 ± 0.1	105
Day 2	21 March	8:30	8.5	8.1	7.1	7.2	7.7 ± 0.7	242
		14:15	27.6	28.1	25.6	27.2	27.1 ± 1.1	1,004
		16:15	21.2	21.4	22.7	22	21.8 ± 0.7	446
Day 3	22 March	8:45	6.5	6.4	5.7	5.7	6.1 ± 0.4	202
		14:00	27.6	27.7	28.1	28.3	27.9 ± 0.3	936
		17:15	13.6	13.1	13.1	13	13.2 ± 0.3	108
Day 4	23 March	10:00	16.2	16.4	16.1	16.2	16.2 ± 0.1	366
		14:00	23.2	23.2	23.1	23.1	23.2 ± 0.1	233
Day 5	24 March	10:00	20.9	20.5	20.4	19.9	20.4 ± 0.4	763
		14:00	33.1	32.8	34.4	33.4	33.4 ± 0.7	815
		17:00	18.2	18.6	17.9	18.4	18.3 ± 0.3	211
Day 6	25 March	9:40	14.7	15.1	14.7	15.5	15.0 ± 0.4	688

*Temperature was measured in each treatment (see Table 1 for growth media abbreviation explanations) and mean ± SD were calculated. Photosynthetically active radiation (PAR) was measured on the surface of the polythene cover*.

#### Algal primary production

The μ_part_ varied every day; the growth rate ranged from −0.05 to 0.89 day^−1^ and from −0.17 to 0.56 day^−1^ in the control and nutrient manipulated treatments, respectively (Table 5). The high variability of μ_part_ was probably caused by weather conditions as indicated by decreased μ_part_ in all cultures at the same time. However, no statistically significant correlations were found between the growth rates of the control and/or the nutrient manipulation treatments and environmental variables (Table 6). A longer cultivation and dataloggers positioned inside the cultivation unit should reveal the response time of the culture to condition changes.

**Table 5:** **μ_part_ and μ (day^−1^; mean ± SD, *n* = 6) of *Chlorella mirabilis* during the out-door experiment (March 19–25, 2005) in control and nutrient manipulation treatments (see Table 1 for growth media abbreviation explanations)**.

	Growth medium			
	Z	ZG	ZGN	ZGC
μ_part 0–1_	0.48 ± 0.24^a^	0.56 ± 0.18^a^	0.36 ± 0.14^a^	0.51 ± 0.09^a^
μ_part 1–2_	−0.05 ± 0.19^b^	−0.17 ± 0.08^b^	−0.12 ± 0.11^b^	−0.03 ± 0.13^b^
μ_part 2–3_	0.32 ± 0.12^ac^	0.45 ± 0.09^a^	0.46 ± 0.13^a^	0.37 ± 0.09^a^
μ_part 3–4_	−0.01 ± 0.13^b^	0.00 ± 0.10^bc^	−0.09 ± 0.07^b^	−0.07 ± 0.11^b^
μ_part 4–5_	0.06 ± 0.12^bc^	0.12 ± 0.12^c^	0.33 ± 0.08^a^	0.36 ± 0.11^a^
μ_part 5–6_	0.89 ± 0.22^d^	0.44 ± 0.04^a^	0.46 ± 0.05^a^	0.52 ± 0.06^a^
μ	0.47 ± 0.07	0.28 ± 0.04	0.39 ± 0.02	0.44 ± 0.03

*The μ_part_ was calculated for each period between subsequent *A*_750_ measurements (μ_part 0–1_ for day 0–day 1; μ_part 1–2_ for day 1–day 2; μ_part 2–3_ for day 2–day 3; μ_part 3–4_ for day 3–day 4; μ_part 4–5_ for day 4–day 5; μ_part 5–6_ for day 5–day 6) and μ for the whole cultivation lasting 8 days. The same letter in superscript indicates homologous groups as recognized by the Tukey HSD test at *p* = 0.05 (*n* = 6 in each group)*.

**Table 6:** **Correlations between environmental conditions measured by AWS and/or in the out-door unit, and μ_part_ of control and nutrient manipulated treatments in the out-door experiment**.

	μ_Z_	μ_ZG_	μ_ZGN_	μ_ZGC_
AWS *T*_mn_ D	0.6091	0.3226	0.5082	0.5221
	*p* = 0.199	*p* = 0.533	*p* = 0.303	*p* = 0.288
AWS *T*_min_ D	0.5000	0.5648	0.4683	0.6841
	*p* = 0.313	p = 0.243	*p* = 0.349	*p* = 0.134
AWS *T*_max_ D	0.7309	0.4245	0.5842	0.6141
	*p* = 0.099	*p* = 0.401	*p* = 0.223	*p* = 0.195
AWS *I*_mn_	−0.1366	−0.4041	−0.3859	−0.5554
	*p* = 0.796	*p* = 0.427	*p* = 0.450	*p* = 0.253
AWS *I*_sum_	−0.1546	−0.4259	−0.3880	−0.5617
	*p* = 0.770	*p* = 0.400	*p* = 0.447	*p* = 0.246
AWS RH D	0.2887	0.3580	0.2404	0.4831
	*p* = 0.579	*p* = 0.486	*p* = 0.646	*p* = 0.332
AWS AP D	−0.1122	−0.1018	−0.1461	0.0177
	*p* = 0.832	*p* = 0.848	*p* = 0.782	*p* = 0.974
AWS *T*_mn_ N	0.3239	0.0378	0.3214	0.2884
	*p* = 0.531	*p* = 0.943	*p* = 0.534	*p* = 0.579
AWS *T*_min_ N	0.1814	−0.0320	0.1813	0.1227
	*p* = 0.731	*p* = 0.952	*p* = 0.731	*p* = 0.817
AWS *T*_max_ N	0.4250	0.0401	0.3428	0.2829
	*p* = 0.401	*p* = 0.940	*p* = 0.506	*p* = 0.587
AWS RH N	0.0073	−0.0546	−0.1784	0.0373
	*p* = 0.989	*p* = 0.918	*p* = 0.735	*p* = 0.944
AWS AP N	0.0378	0.3418	0.1002	0.2902
	*p* = 0.943	*p* = 0.507	*p* = 0.850	*p* = 0.577
Micro *T*_mn_	0.3149	0−0.0399	0.3116	0.1658
	*p* = 0.543	*p* = 0.940	*p* = 0.548	*p* = 0.754
Micro PAR_mn_	0.2335	−0.1034	0.0468	−0.1315
	*p* = 0.656	*p* = 0.845	*p* = 0.930	*p* = 0.804

*AP, air pressure; AWS, data measured by AWS; D, day time (sunrise to sunset); *I*_mn_, mean irradiance; *I*_sum_, sum or received irradiance; Micro, data measures in the out-door cultivation unit; μ, growth rate, lower index indicates treatment (see Table 1 for treatment abbreviations); N, night time (sunset to sunrise); PAR_mn_, mean PAR; RH, air humidity; *T*_max_, maximum temperature; *T*_min_, minimum temperature; *T*_mn_, mean temperature*.

Several freezing periods were recorded during the out-door cultivation (Figure 4) that could affect the growth rate of the cultures. The cultures were frozen during the first three nights of the experiment. Microscopic examination of the cells after thawing of the cultures revealed destruction of about 30% of the cells after every bout of freezing. The culture in normal composition Z medium exhibited complete freezing whenever the temperature dropped below –8°C. The cultures in ZG and ZGC remained in a semi-frozen state while ZGN remained in a liquid phase down to –15°C.

**Figure 5:**
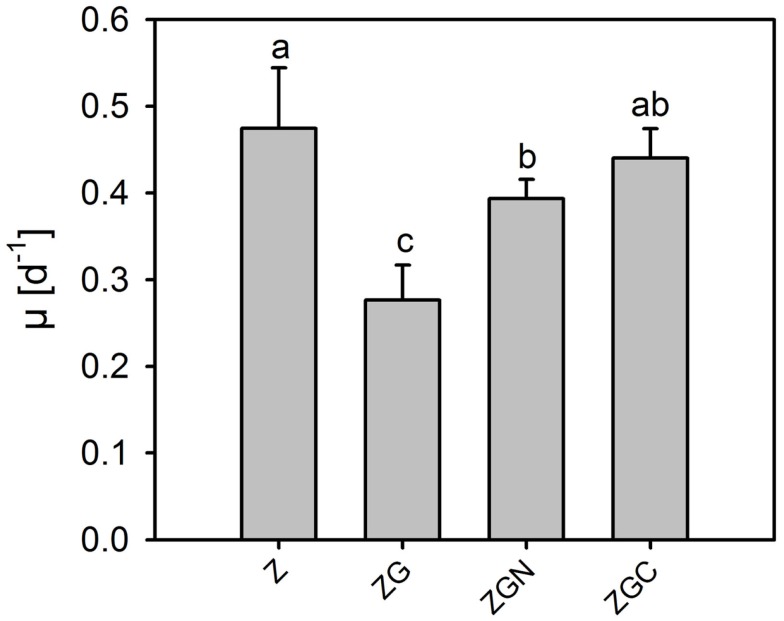
**Relative growth rates of *Chlorella mirabilis* under different nutrient treatments in the out-door micro-scale mass cultivation experiment**. See Table 1 for treatment abbreviations (mean ± SD, *n* = 6). The same letter indicates homologous groups as recognized by the Tukey HSD test at *p* = 0.05; the Z medium (control) is included in the group labeled “a.”

Means in the out-door experiment were higher than those in the in-door experiment for the control as well as every nutrient manipulated treatments (*t*-test, *n* = 6 in each treatment, *p* < 0.049 in all cases, Tables 2 and 5; Figures 2 and 5). The μ of the control ranged from 0.41 to 0.61 day^−1^, which is about four times higher than in the in-door experiment. The growth rates of the nutrient manipulated treatments ranged from 0.21 to 0.48 day^−1^, about twice that in the in-door cultivation. The increased growth rates in the out-door cultivation could be caused by several factors:

(a) increased temperature, especially during the light phases,
(b) higher amount of received light energy (1,382 W m^−2^ during in-door experiment vs. 2,578–21,984 W m^−2^ during the out-door experiment per day; data from in-door cultivation were recalculated according to formulas of Thimijan and Heins (1983) and
(c) different air-liquid interface area affecting CO_2_ diffusion into the medium due to different types of cultivation vessels.

**Figure 6:**
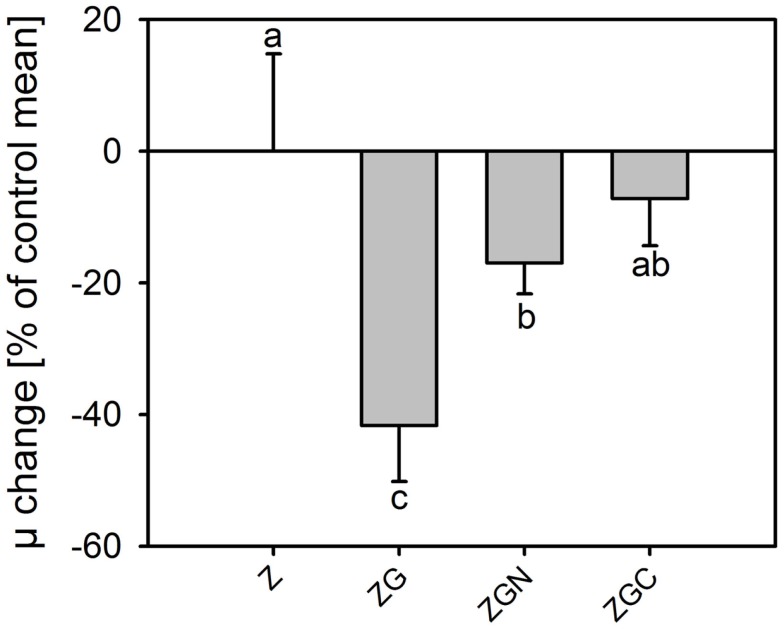
**Change in relative growth rates of *Chlorella mirabilis* under different nutrient treatments in the out-door micro-scale mass cultivation experiment**. The values were normalized to the control mean (100%). The zero line corresponds to 100% of the control. See Table 1 for treatment abbreviations (mean ± SD, *n* = 6). The same letter indicates homologous groups as recognized by the Tukey HSD test at *p* = 0.05; the Z medium (control) is included in the group labeled “a.”

Contrary to the in-door cultivation, nutrient addition decreased the growth rate (*t*-test, *n* = 6 in each treatment, *p* < 0.002; Figure 5). The most profound growth inhibition, by 42 ± 8% compared to the control, was observed in the ZG treatment (Figure 6). Surprisingly, glycerol reduced the growth rate significantly (HSD for non-equal *n*, *p* = 0.026, Figure 6). Effects of the other nutrient additions were not significant.

### Fatty acids composition

The fatty acid analyses revealed increased production of linoleic acid in glycerol treatments. The fatty acid composition was similar in both experiments for individual treatments; however it differed among the treatments (Table 7). Oleic acid 18:1 (*n*−9) was the most abundant fatty acid found in the biomass of *Chlorella mirabilis* cultivated in the in-door experiment. This fatty acid can be found in all lipids of animal and plant origin. The *trans*-isomer of oleic acid (elaidic acid) is rarely found and also in this case it comprised only ca. 5% of the oleic acid content. Linoleic acid 18:2 (*n*−6) is an essential fatty acid in animal diets, because it is necessary for growth, reproduction and healthy development. It is the precursor of a family of other fatty acids. Its content in the biomass was about 11% of that of the oleic acid. There were some saturated acids in the biomass, e.g., palmitic acid 16:0 and myristic acid 14:0. When glycerol was added to the medium, the content of linoleic acid increased to 51% of the oleic acid content. The addition of 50 μM KNO_3_ had no effect regarding production of unsaturated fatty acids. On the contrary, the content of saturated palmitic acid increased.

**Table 7:** **Fatty acid composition in the in-door and out-door cultivations for different treatments**.

Sample	Fatty acids composition (%)					
	Myristic acid	Palmitic acid	Linoleic acid	Oleic acid	Elaidic acid	Unidentified
	14:0	16:0	18:2 (*n*−6)	18:1 (*n*−9)	18:1 (*n*−9)	
**IN-DOOR EXPERIMENTS**						
Z	1.70	7.83	7.70	70.44	1.97	10.36
ZG	0.37	16.00	22.60	40.31	1.62	19.10
ZGN	1.49	23.46	15.13	49.97	2.83	7.12
**OUT-DOOR EXPERIMENTS**						
Z	0.87	10.72	16.86	49.87	0.83	20.85
ZG	1.55	27.03	9.98	49.62	3.65	8.17
ZGN	1.44	23.84	10.20	43.28	10.37	10.87

*See Table 1 for treatment abbreviations*.

## Discussion

Manipulation of culture conditions to enhance growth and production of useful compounds is one of the pre-requisites in micro-algal biotechnology (Dunstan et al., 1994). There are numerous reports about the enhancement of growth and biomass yield by altering pH, aeration rate, irradiance, temperature, cell concentration, and nitrogen and carbon sources in the medium (Cohen et al., 1988; Borowitzka, 1997) in strains of tropical, subtropical, and temperate origins. There is substantial data in the literature to support the fact that biomass yield of a few strains of micro-algae can be significantly increased by subjecting the cultures to mixotrophic growth conditions (Samejima and Myers, 1958). However, information is still scarce about polar strains of micro-algae with respect to culture conditions to enhance growth and production of useful compounds.

Our in-door cultivation experiments indicated that the Antarctic soil strain of *Chlorella mirabilis* shows an affinity toward mixotrophy. This was evident from the faster growth rate in the presence of 5% glycerol as compared to purely photoautotrophic cultures. These observations are in agreement with earlier reports for *Chlorella sorokiniana* (Lee and Low, 1992), *Spirulina platensis* (Chen and Zhang, 1997) and the diatom *Phaeodactylum tricornutum* (Cerón Garcí et al., 2000).

Results of the in-door experiment also suggest that our soil strain of *Chlorella mirabilis* has developed the ability to utilize organic compounds in general, because of the fact that the Antarctic soil environment contains a wide spectrum of diluted organic substances and also polar summer light conditions are very variable. The Antarctic habitat, with a gradual accumulation of organic matter (due to slower mineralization processes), seems to be another logical explanation for the evolution of mixotrophy in polar soil micro-algae.

As has been shown in various reports, the lack of inorganic carbon and nitrogen often limits primary production in polar terrestrial and freshwater habitats (Henry and Svoboda, 1986; Davey and Rothery, 1992; Liengen and Olsen, 1997; Elster and Komárek, 2003; Walker et al., 2008). Our in-door cultivation experiment showed a positive response under additions of nitrogen and inorganic carbon.

Our analyses have proven that organic carbon (glycerol) is probably the key parameter influencing the growth rate of the Antarctic strain of *Chlorella mirabilis*. Growth rate responses to the addition of inorganic carbon and nitrogen were negligible or negative and could be omitted in low-temperature mass cultivation of this strain. However, growth rate responses to the addition of organic or inorganic carbon and nitrogen are strain-specific. In our previous study (Shukla et al., 2011), it was shown that, for a majority of polar *Chlorella*-like strains, the addition of inorganic nitrogen (in both N-NO_3_ and N-NH_4_ forms) rather inhibited the growth rate indicating thus possible nitrogen oversaturation. The positive effect of carbon addition observed by Shukla et al. (2011) could indicate carbon limitation during the experiment.

The growth rates in the out-door cultivation were generally higher than for the in-door conditions. There are several explanations for these findings:

(a) increased temperature of up to 23°C in the out-door cultivation stimulated micro-algal growth. Under natural conditions of Trebon, Czech Republic, Central Europe (during late winter and/or early spring 2005), the growth of the soil Antarctic strain *Chlorella mirabilis* dramatically increased during the period when light and temperature fluctuated between 250 and 1,000 μmol m^−2^ s^−1^ and 8–23°C. On the contrary, growth was arrested during and after the intermittent freezing of the cultures after a sudden drop in the atmospheric temperature. Microscopic examination of the cells after thawing of the cultures revealed destruction of about 30% of the cells after every bout of freezing. The culture in normal composition Z medium exhibited complete freezing whenever the temperature dropped below –8°C. The cultures in ZG and ZGC remained in a semi-frozen state while ZGN remained in a liquid phase down to –15°C. This observation suggests that modification of the growth medium (Z medium) with 5% glycerol, and inorganic carbon and nitrogen sources can serve two purposes at the same time: (i) glycerol can prevent freezing of a culture thereby preventing loss of viable cells during intermittent freezing and (ii) it can support mixotrophic growth. It was observed during the experiments that a heating device is indispensable for out-door cultivation during early spring. A controlled heating of cultures during the period when the temperature drops below 0°C can prevent the loss of cells due to lysis during freezing and therefore supports a better yield of biomass.
(b) the amount of received light energy per day was up to 10 times higher in the out-door cultivation than in the in-door experiment. The observed irradiance values were high enough to cause photoinhibition, however the micro-algal suspension was exposed to such high PAR for a relatively short time. Moreover, suspension mixing can provide additional protection against excessive PAR (Oliver et al., 2003). Continuous monitoring of photosynthetic performance using variable chlorophyll fluorescence instrumentation will provide additional information on light utilization as has been proposed by Torzillo et al. (1996), Torzillo et al. (1998), and Torzillo et al. (2003) for mass cultivation control.
(c) CO_2_ supply could be affected by the air-liquid interface area, which was larger in the out-door experiment. The larger area provided increased input of CO_2_ and, together with higher temperature and irradiance, led to higher growth rates.

Since there were no dataloggers inside the out-door cultivation unit, meteorological data serve as a good proxy for microclimate conditions. Mathematic models based on meteorological data and cultivation unit properties are proposed to be used for prediction of microclimate conditions in mass cultivation. Thus, short-term weather forecasts should be used for mass cultivation planning and operation.

We have proposed to use 5% glycerol in out-door mass cultivation of polar strain of *Chlorella mirabilis* in conditions where temperature fluctuates widely (up to −15°C). In dark or low light conditions, glycerol can support mixotrophy in micro-algal cultivation (Faust and Gantt, 1973), however glycerol utilization by *Chlorella*-like species strains seems to be strain- or species-specific. No glycerol utilization was reported for *Chlorella vulgaris* (Liang et al., 2009). The inhibitory effect of glycerol in concentrations above 1% (v/v) was observed for this strain. However, in *Chlorella protothekoides* mass cultivation, glycerol addition has been used for stimulation of fatty acid production (O’Grady and Morgan, 2011). Species- and/or strain-specific reactions also influence the response to glycerol treatment, as was observed in other algal toxicity assays using different algal strains (Blanck et al., 1984; Lukavský et al., 2003). Generally, a negative or positive glycerol effect on growth rate could be influenced by environmental conditions, especially light as seen in other bioassays (Mayasich et al., 1987; Mayer et al., 1998; Cleuvers et al., 2002). Even a relatively small change in light conditions can lead to a different response.

Increased production of unsaturated fatty acids is a general response to low-temperature conditions (Satoh et al., 1979; Gombos et al., 1992; Hu et al., 2008; řezanka et al., 2008, 2009). The increase was indeed observed in the in-door experiment for linoleic and elaidic acids. In the out-door experiment, the expected increase in unsaturated fatty acids content was not observed, with the exception of elaidic acid. These data indicate that the temperature conditions were not limiting for the used strains and specific adjustments of membrane fatty acids composition was not necessary for survival. In the in-door experiment, glycerol stimulated production of linoleic acid only. Such increased production of fatty acids stimulated by glycerol was observed in micro-algae (Wood et al., 1999), but have not been reported for *Chlorella* strains yet. The effect of glycerol addition was probably caused by different cultivation conditions as mentioned above.

## Conclusions

The study showed that the biomass yield of polar soil micro-algae in mass-culture units under natural conditions of temperate/polar regions can be significantly improved by appropriate manipulation of the growth medium.

The used micro-algal strain *Chlorella mirabilis* Lukešová 10/1997 could be a valuable source of linoleic acid, since increased production was detected in glycerol treatments in both the in-door and out-door mass cultivations. Therefore, this should be considered as a perspective strain for linoleic acid production in low-temperature biotechnologies.

## Conflict of Interest Statement

The authors declare that the research was conducted in the absence of any commercial or financial relationships that could be construed as a potential conflict of interest.
